# Prevalence of Quinolone Resistance of Extended-Spectrum β-Lactamase-Producing *Escherichia coli* with ST131-*fimH30* in a City Hospital in Hyogo, Japan

**DOI:** 10.3390/ijms20205162

**Published:** 2019-10-18

**Authors:** Masazumi Teramae, Kayo Osawa, Katsumi Shigemura, Koichi Kitagawa, Toshiro Shirakawa, Masato Fujisawa, Takayuki Miyara

**Affiliations:** 1 Department of Biophysics, Kobe University Graduate School of Health Sciences, Kobe 6540142, Japan; masa_terara84@hotmail.com; 2 Department of Clinical Laboratory, Hyogo Prefectural Awaji Medical Center, Awaji 6560021, Japan; 3 Department of Infection Prevention and Control, Kobe University Hospital, Kobe 6540142, Japan; miyarat@med.kobe-u.ac.jp; 4 Division of Health Science, Department of Medical Technology, Kobe Tokiwa University, Kobe 6530838, Japan; 5 Division of Infectious diseases, Department of Public Health, Kobe University Graduate School of Health Sciences, Kobe 6540142, Japan; katsumi@med.kobe-u.ac.jp (K.S.); ko1.kitgwa@gmail.com (K.K.); 6 Division of Urology, Department of Organ Therapeutics, Faculty of Medicine, Kobe University Graduate School of Medicine, Kobe 6500017, Japan; toshiro@med.kobe-u.ac.jp (T.S.); masato@med.kobe-u.ac.jp (M.F.); 7 Division of Translational Research for Biologics, Department of Internal Medicine Related, Kobe University Graduate School of Medicine, Kobe 6578501, Japan

**Keywords:** *Escherichia coli*, ESBL, ST131, *fimH*, CH typing

## Abstract

Extended-spectrum β-lactamase (ESBL)-producing *Escherichia coli* isolates are known to tolerate superior quinolone antimicrobials compared with other antibacterial agents. Among the clones belonging to sequence type (ST) 131 by multilocus sequence typing, the involvement of the H30-Rx subclone has been reported worldwide with various *fimH* genes encoding type 1 pili. We investigated 83 isolates of ESBL-producing *E. coli* and performed antimicrobial susceptibility test, CH (*fumC*/*fimH*) ST131 by typing the specific PCR. Moreover, mutation analysis of genes involved in quinolone antibiotic resistance (*gyrA* and *parC*) and ESBL genotypes were determined. As a result, 54 of 83 isolates (65.1%) of CH40-30 clones corresponding to ST131-*fimH30* were detected, and all were resistant to levofloxacin. Mutations associated with this resistance were common, and included S83L and D87N of *gyrA* and S80I and E84V of *parC*. Subclone analysis revealed a high proportion of *fimH30*-non-Rx (40 isolates, 74.1%). Each subclone was characterized by ESBL genotype, and the CTX-M-15 type was mainly seen for *fimH30*-Rx, with the CTX-M-14 type or CTX-M-27 type seen for *fimH30*-non-Rx. This study suggests that an increase in ESBL-producing quinolone-resistant *E. coli* in a city hospital in Hyogo, Japan, was caused by the spread of subclones belonging to *fimH30*-non-Rx of ST131.

## 1. Introduction

*Escherichia coli* is a causative microorganism of infectious diseases, such as those of the urinary tract and bloodstream. However, since the mid-2000s, resistance to β-lactam antibiotics has been reported to be increasing worldwide [1]. According to research by Japan Nosocomial Infections Surveillance, the resistance rate of *E. coli* to cefotaxime (CTX) in Japan was 9% in 2008 and reached 26% in 2016 [2]. The main antimicrobial resistance mechanism of *E. coli* is the inactivation of antimicrobials by isolates with extended-spectrum β-lactamase (ESBL) production. ESBL genotypes include TEM and SHV types, but those most widely spread around the world are CTX-M types. Amino acid sequence homology roughly divides these into five classes, of which CTX-M-1, 2, and 9 are the major groups [3,4]. The resulting rise in infection caused by the escalation of ESBL-producing bacteria has necessitated an increase in the use of carbapenems, which are first-line drugs, but are treated as final choices because of their broad spectrum; this, therefore, increases the risk of developing further resistant bacterial isolates [5].

One of the main clones isolated in the worldwide spread of ESBL-producing *E. coli* is ST131 [6]. *E. coli* is also classified into four major phylogenetic groups (A, B1, B2, and D) by phylogenetic analysis. *E. coli* ST131 belongs to B2, which is more pathogenic than the other groups, and many isolates are characterized by serotype O25b [7,8]. *E. coli* ST131 has been isolated worldwide since the report in 2008 and accounted for 52% of ESBL-producing *E. coli* in a Japanese survey conducted from 2008–2011 [8,9].

ESBL-producing *E. coli* shows increased resistance to quinolone antibiotics compared with non-producing isolates [1,10,11,12,13,14,15]. Therefore, with the documented increase of ESBL-producing isolates, the resistance rate of levofloxacin (LVFX) increased from 27% in 2008 to 39% in 2016 [2]. The target enzymes of quinolone antibacterial drugs are DNA gyrase and topoisomerase IV, which convert the three-dimensional structure of DNA, but amino acid substitutions are reported for the quinolone resistance-determining regions (QRDRs) of *gyrA* and *parC*, encoding DNA gyrase and topoisomerase IV, respectively [16].

Clones belonging to *fimH30* are important for *fimH* coding of type 1 pili attachment among ST131 as a background for simultaneous resistance to these drugs with different mechanisms of action/resistance [8,17]. *fimH30*-Rx as the *fimH30* subclone contains a number of single nucleotide polymorphisms (SNPs) in the core genome [18]. There are concerns that *E. coli* ST131-*fimH30-Rx* is increasing worldwide [11,12,13,14]. Moreover, the combination of ST and *fimH* enables a detailed evaluation of clones. Therefore, the usefulness of CH typing by a combination of the *fumC* allele number and *fimH* type used for Multilocus sequence typing (MLST) was investigated [19].

The characteristics of ESBL-producing *E. coli* are of major importance in terms of public health. They are some of the most common resistant bacteria, but Japanese data of bacterial clones, especially *fimH*, are insufficient and region-dependent. Therefore, the present study investigated the epidemic status of the ST131-*fimH30* subclone and its involvement in quinolone antibiotic resistance in ESBL-producing *E. coli* using repetitive-sequence-based PCR (rep-PCR) as an epidemiological method [15] in a city hospital in Hyogo Prefecture, Japan.

## 2. Results

### 2.1. CH Typing

Fifteen types were detected by CH typing, with the most frequent being CH 40-30 in 54 of 83 isolates (65.1%). Subsequently, six isolates (7.2%) were identified as CH 14-64, five (6.0%) as CH 40-41, and three (3.6%) as CH 26-5 and CH 100-96, and others (*n* = 12, 14.5%).

Phylogenetic analysis classified 69 isolates (83.1%) as B2, 13 isolates (15.7%) as D, and one isolate (1.2%) as B1. A total of 65 isolates of CH40-30, CH14-64, and CH40-41 consisting of five or more isolates belonged to B2, whereas 13 of the remaining 18 (72.2%) were classified as D. Fifty-three isolates (63.9%) belonging to CH40-30 showed serotype O25b.

ST131-specific PCR revealed that all clones with 40 alleles of *fumC* were ST131 (*n* = 60, 72.3%). All of CH40-30 (*n* = 54) corresponded to ST131-*fimH30*, CH40-41 (*n* = 5) was ST131-*fimH41* and CH40-89 was ST131-*fimH89* (*n* = 1). Of the 54 ST131-*fimH30* isolates, 14 (25.9%) were *fimH30-Rx*, accounting for 17% of the total, and the remaining 40 (74.1%) *fimH30*-non-Rx isolates accounted for 48.2% of the total 83 isolates.

### 2.2. Antimicrobial Resistance

Antimicrobial susceptibility test results are shown in Table 1. Sixty-six (79.5%) of the 83 isolates showed resistance to LVFX. By CH type, 54 isolates of CH40-30 and six isolates of CH14-64 were resistant to LVFX, whereas all five isolates of CH40-41 were sensitive to LVFX.

For antibacterial drugs other than quinolones, a comparison of CH40-30 with other CH types showed no difference in antimicrobial resistance to β-lactam antibiotics, but significant GM and SXT sensitivity was detected (*p* < 0.01 and *p* < 0.001, respectively). Five (83.3%) of six isolates with CH14-64 showed resistance against SXT.

### 2.3. Antimicrobial Resistance Genes

Amino acid analysis of QRDRs focused on 83Ser (S) and 87Asp (D) in *gyrA* and 80Ser (S) and 84Glu (E) in *parC*. All mutations were substitutions to different amino acids, 83Leu (L), and 87Asn (N) or Tyr (Y) in *gyrA,* and 80Ile (I) and 84Val (V) in *parC*, and six combinations of each were observed. A total of 54 isolates (65.1%) of LNIV representing S83L and D87N of *gyrA*, and S80I and E84V of *parC*, followed by 10 isolates (12.0%) of LNIE representing S83L and D87N of *gyrA*, and S80I and E84 of *parC*, nine isolates (10.8%) of LDSE representing S83L and D87 of *gyrA*, and S80 and E84 of *parC*, and seven isolates (8.4%) had no mutations (Table 2). Among the four sites, a total of three or more substitutions were associated with LVFX resistance. With the CH type, CH 40-30 was shown to be LNIV, CH 14-64 was LVFX-resistant and closely related to LNIE, and CH 40-41 was LVFX-sensitive but related to LDSE.

For ESBL, all 83 isolates were classified as CTX-M type, in either the CTX-M-1 group, CTX-M-2 group, or CTX-M-9 group. Sequencing showed that the most common genotype was CTX-M-14 belonging to the CTX-M-9 group, which was seen in 38 isolates (45.8%). Seventeen isolates (20.5%) of CTX-M-27 belonged to the CTX-M-9 group followed by 16 isolates (19.3%) of CTX-M-15 in the CTX-M-1 group. The remaining CTX-M types were: 5 isolates (6.0%) of CTX-M-2, 5 isolates (6.0%) of CTX-M-55 and 1 isolate (1.2%) of CTX-M-3 belonged to the CTX-M-1 group, and 1 isolate (1.2%) having both CTX-M-15 and CTX-M-27 (Supplemental Table S1). Table 3 shows the CTX-M types of ST131-*fimH30*-Rx and -non-Rx belonging to CH40-30. All 14 isolates of ST131-*fimH30*-Rx were CTX-M-15. Of the 40 isolates of ST131*-fimH30*-non-Rx, 26 were CTX-M-14 (65.0%), 12 were CTX-M-27 (30.0%), and one was CTX-M-15 (2.5%). One isolate (2.5%) belonging to ST131-*fimH30*-non-Rx carried CTX-M-15 and CTX-M-27 type ESBL-producing genes.

### 2.4. Rep.-PCR

Typing using rep-PCR was divided into 14 groups (A to N) (Supplemental Figure S1). Almost all isolates (*n* = 57) of ST131, including 51 isolates of ST131-fimH30 (CH40-30) and 1 isolate of non ST131, belonged to the H group.

## 3. Discussion

The epidemic spread of ESBL-producing isolates is thought to be reflected in the quinolone resistance rate. It is reported that quinolone-resistant ST131-*fimH30* has spread before the acquisition of ESBL-producing genes as a reason why various ESBL-producing genes are found in quinolone-resistant clones [1]. Especially, *fimH30* subclone *fimH30*-Rx is characterized by CTX-M-15-type ESBL production of the CTX-M-1 group, with specific amino acid substitutions (Ser83Leu and Asp87Asn in *gyrA*, and Ser80Ile and Glu84Val in *parC*) in QRDRs of quinolone resistance.

The *fimH30* subclone *fimH30*-Rx has mainly been reported outside Japan [1,10,11,12,13]. Factors responsible for the quinolone resistance of ESBL-producing *E. coli* were investigated for relevance to clones using CH (*fimC*/*fimH*) typing. In the present study, CH40-30, 40-41, and 14-64, consisting of five or more isolates, each correlated with the antimicrobial sensitivity result of LVFX, and the amino acid mutation pattern in QRDRs was the same for each CH type. Among them, CH40-30, which demonstrates LVFX resistance, accounted for 65% of ESBL-producing isolates. CH typing has a better clone distinguishing ability than MLST alone and can predict ST more accurately. In the case of ST131, the corresponding *fumC* allele number was 40; conversely, if it was CH 40-30, the possibility of ST 131-*fim H30* can be deemed high. CH 40-30 isolates also tended to have lower resistance to GM and SXT than other CH types, regarding drug sensitivity other than quinolone and β-lactam antibiotics. For CHs other than CH 40-30 isolates, individual evaluation was difficult because of limited isolation, but CH 14-64 isolates were shown to be resistant to SXT and five of six isolates were resistant to LVFX, while all five isolates of CH40-41 were sensitive to LVFX. CH14-64 isolates mainly corresponded to ST1193, whereas these clones were shown with the resistance of SXT and tetracycline in addition to quinolone resistance were reported to increase in the United States [12].

All 54 isolates of CH40-30 were ST131-*fimH30*, belonging to the highly pathogenic phylogenetic group B2, and almost all isolates belonged to the same group by rep-PCR. Additionally, 53 isolates were of serotype O25b, whereas ESBL-producing genes showed multiple CTX-M types. H30-Rx isolates with CTX-M-15 type ESBL production have been recognized as worldwide pandemic clones, especially in Europe and the United States, but it is also the most common ESBL-producing clone in ST131 in Korea [13]. Instead, *fimH30*-non-Rx, which produces CTX-M-14 or CTX-M-27 ESBL, accounted for almost half of isolates, suggesting a difference from the global trend. Our results observed an increased number of clones characterized by CTX-M-27-type ESBL production among ST131-*fimH30*-non-Rx and a decrease in the CTX-M-14 type compared with the result of a previous study in Kyoto and Shiga [14]. Those authors reported separation rates of CTX-M-27 and CTX-M-14 types of 45% and 24%, respectively, from 2008 to 2012. ESBL-producing isolates with the genotype of the CTX-M-9 group are more commonly isolated in Japan compared with the CTX-M-15 type [4,15,20]. In this survey, the isolation rate of CTX-M-14 type ESBL-producing bacteria was still high, and regional differences were confirmed, even in Japan.

In a Japanese epidemiological survey, ST131-*fimH30* was the most dominant type of *E. coli* (including ESBL non-producing isolates) isolated from an acute phase hospital in 2014, accounting for 25% of the total [21]. Its high prevalence in ESBL-producing *E. coli* has not yet been clarified in Japan.

The strong association between ST131-*fimH30*-Rx and CTX-M-15 suggests a bias towards ESBL genotypes that are easy for each clone to acquire. In an American study, the separation rate of ST131-*fimH30* was higher in hospitalized patients than in the general community and increased after the age of 30. This indicates that clones adapt to medical facility environments and thus may spread among patients [22]. To evaluate this in the present case, it will be necessary to clarify the epidemic status of the ST131-*fimH30* subclone targeting non-ESBL producing isolates, which should be done over time. However, the limitation of this research was that the stock was separated over a limited period in 2016 within a single hospital, and so it may not be possible to fully evaluate the trend.

In conclusion, the subclone *fimH30*-non-Rx was involved in the spread of quinolone-resistant ESBL-producing *E. coli* in a city hospital in Hyogo Prefecture. Specifically, it was ST131-*fimH30*, which had distinct resistance mechanisms. To clarify the cause of the spread, it will be necessary to expand the target of surveillance focusing on ST131-*fimH30* and non-ESBL producers, and to continue investigations in the future.

## 4. Materials and Methods

### 4.1. Isolates

*E. coli* isolates were separated from clinical specimens in Hyogo Prefectural Awaji Medical Center between April 2016 and March 2017. These isolates were collected from urine (52 (62.7%)), blood (11 (13.3%)), sputum (7 (8.4%)), wound (4 (4.8%)), bile (3 (3.6%)), abdominal dropsy (2 (2.4%)), pus (3 (3.6%)), and pleural effusion (1 (1.2%)). Of the *E. coli* isolates identified, 83 showed resistance to CTX or ceftazidime (CAZ), and were determined to be ESBL-positive using the double disk synergy test confirming the effect of clavulanic acid on ESBL [23]. Isolate collection avoided duplications from the same patient.

### 4.2. Antimicrobial Susceptibility Test

We measured ampicillin-sulbactam (ABPC/SBT), piperacillin–tazobactam (PIPC/TAZ), cefmetazole (CMZ), CTX, CAZ, cefepime (CFPM), Aztreonam (AZT), LVFX, gentamicin (GM), and sulfamethoxazole–trimethoprim (SXT) by minimum inhibitory concentrations (mg/L) with the microdilution method using the NENC 1 J panel (Beckman Coulter, Tokyo, Japan). Criteria were in accordance with Clinical and Laboratory Standards Institute M 100-S22 [24], although intermediate criteria were included for resistance.

### 4.3. CH (fumC/fimH) Typing

For ESBL-producing *E. coli*, CH (*fumC* / *fimH*) typing based on a combination of the *fumC* allele number and *fimH* type used for MLST was typically used [19]. ST131 was determined by detecting ST131-specific SNPs of *mdh* and *gyrB* used in the Achtman scheme of MLST [25]. *fimH* compares the base sequence obtained from the online database and classifies it by type. For isolates classified as *fimH30*, H30-Rx was determined by specific SNP detection of *ybbW* [26,27].

The phylogenetic group detected three genes (*chuA*, *yjaA*, and *TspE4.C2*) by PCR and classified them as A, B1, B2, or D groups [7]. For serotypes, O25b was determined using specific PCR [28].

### 4.4. QRDR Analysis

To identify mutations associated with quinolone resistance, the nucleotide sequence of QRDRs in *gyrA* and *parC* was determined by sequencing and compared with wild-type *E. coli* (K-12 MG 1655 isolate, GenBank accession number NC000913) by BLAST (https://blast.ncbi.nlm.nih.gov/Blast.cgi, accessed on 23 December 2018). Amino acid substitutions were also examined [29].

### 4.5. ESBL Genotyping

The detection of CTX-M-1, CTX-M-2, and CTX-M-9 groups was carried out by multiplex PCR as previously described [20]. ESBL-producing genes classified into CTX-M-1 and CTX-M-9 groups by sequencing to determine the detailed genotype.

### 4.6. Repetitive-Sequence-Based PCR

DNA was extracted using the UltraClean microbial DNA isolation kit (bioMérieux, Marcy l’Etoile, France) and rep-PCR was performed with the DiversiLab Escherichia kit (bioMérieux). The products were detected by the Agilent analyzed 2100 Bioanalyzer (Agilent Technologies, Inc., Palo Alto, CA) and the results were analyzed by Diversilab analysis software (ver. 3.4, bioMérieux) as previously described [20].

### 4.7. Statistical Analysis Method

The Chi-squared test was used for statistical analysis, and a difference of 5% or less was deemed significant.

## Figures and Tables

**Table 1:** CH (*fumC*/*fimH*) type and antimicrobial resistance of 83 extended-spectrum β-lactamase (ESBL)-producing *Escherichia coli* isolates.

Antibiotics	Total *n* (%)	CH type ^a^					
		40-30 (*n* = 54)	14-64 (*n* = 6)	40-41 (*n* = 5)	26-5 (*n* = 3)	100-96 (*n* = 3)	Others (*n* = 12)
LVFX	66 (79.5)	54 (100)	6 (100)	0 (0)	1 (33.3)	0 (0)	5 (41.7)
ABPC/SBT	67 (80.7)	42 (77.8)	4 (66.7)	4 (80.0)	3 (100)	3 (100)	11 (91.7)
PIPC/TAZ	2 (2.4)	1 (1.9)	0 (0)	1 (20.0)	0 (0)	0 (0)	0 (0)
CMZ	1 (1.2)	1 (1.9)	0 (0)	0 (0)	0 (0)	0 (0)	0 (0)
CTX	83 (100)	54 (100)	6 (100)	5 (100)	3 (100)	3 (100)	12 (100)
CAZ	38 (45.8)	27 (50.0)	2 (33.3)	2 (40.0)	1 (33.3)	2 (66.7)	4 (33.3)
CFPM	74 (89.2)	48 (88.9)	5 (83.3)	4 (80.0)	3 (100)	3 (100)	11 (91.7)
AZT	71 (85.5)	47 (87.0)	5 (83.3)	4 (80.0)	3 (100)	2 (66.7)	10 (83.3)
GM	22 (26.5)	9 (16.7) ^b^	3 (50.0)	3 (60.0)	2 (66.7)	1 (33.3)	4 (33.3)
SXT	23 (27.7)	8 (14.8) ^b^	5 (83.3)	1 (20.0)	3 (100)	0 (0)	6 (50.0)

LVFX: Levofloxacin, ABPC/SBT: Ampicillin–Sulbactam, PIPC/TAZ: Piperacillin–Tazobactam, CMZ: Cefmetazole, CTX: Cefotaxime, CAZ: Ceftazidime, CFPM: Cefepime, AZT: Aztreonam, GM: Gentamicin, SXT: Sulfamethoxazole–Trimethoprim; ^a^ *fumC* allele type and *fimH* type combination; ^b^ CH40-30 has a significantly lower GM and SXT resistance rate than other CH types (*p* < 0.01, *p* < 0.001 by chi-square test, respectively).

**Table 2:** Quinolone resistance-determining regions (QRDRs) mutations by CH type in 83 ESBL-producing *E. coli* isolates.

			CH type ^a^					
QRDRs mutations ^b^ (number of mutations)	LVFX susceptibility	Total *n* (%)	40-30 (*n* = 54)	14-64 (*n* = 6)	40-41 (*n* = 5)	26-5 (*n* = 3)	100-96 (*n* = 3)	others (*n* = 12)
SDSE (0)	S	7 (8.4)	0 (0)	0 (0)	0 (0)	0 (0)	3 (100)	4 (33.3)
LDSE (1)	S	9 (10.8)	0 (0)	0 (0)	5 (100)	1 (33.3)	0 (0)	3 (25.0)
LDIE (2)	S	1 (1.2)	0 (0)	0 (0)	0 (0)	1 (33.3)	0 (0)	0 (0)
LYIE (3)	R	1 (1.2)	0 (0)	0 (0)	0 (0)	1 (33.3)	0 (0)	0 (0)
LNIE (3)	R	10 (12.0)	0 (0)	6 (100)	0 (0)	0 (0)	0 (0)	4 (33.3)
LNIG (4)	R	1 (1.2)	0 (0)	0 (0)	0 (0)	0 (0)	0 (0)	1 (8.3)
LNIV (4)	R	54 (65.1)	54 (100)	0 (0)	0 (0)	0 (0)	0 (0)	0 (0)

S: Susceptible, R: Resistant; ^a^ *fumC* allele type and *fimH* type combination; ^b^ Substitution of QRDRs was expressed by amino acids in the order of 83 Ser (S) or Leu (L) and 87 Asp (D) or Asn (N) or Tyr (Y) in *gyrA,* and 80 Ser (S) or Ile (I) and 84 Glu (E) or Val (V) in *parC* from the left (SDSE is wild-type).

**Table 3:** Subclones and CTX-M type of ESBL-producing *E. coli* CH40-30 (ST131-*fimH30*) in 54 isolates.

CTX-M-type	Total *n* (%)	*fimH*30-Rx (*n* = 14)	*fimH*30-non-Rx (*n* = 40)
CTX-M-15	15 (27.8)	14 (100)	1 (2.5)
CTX-M-14	26 (48.1)	0 (0)	26 (65.0)
CTX-M-27	12 (22.2)	0 (0)	12 (30.0)
CTX-M-15+CTX-M-27	1 (1.9)	0 (0)	1 (2.5)

## Supplementary Materials

Supplementary materials can be found at https://www.mdpi.com/1422-0067/20/20/5162/s1. Figure S1: Dendrogram of repetitive-sequencebased PCR typing with 95% simirity level for 83 isolates of extended-spectrum β-lactamase (ESBL)-producing *Escherichia coli.* Sequence type (ST). Table S1: Characteristics of 83 ESBL-producing *E. coli* isolates.
