# Supplementary material for: Prevalence of Quinolone Resistance of Extended-Spectrum β-Lactamase-Producing Escherichia coli with ST131-fimH30 in a City Hospital in Hyogo, Japan
Source: Int J Mol Sci. 2019 Oct 18;20(20):5162. doi: 10.3390/ijms20205162 (PMC6829264; doi:10.3390/ijms20205162)
Supplement: Supplementary file 1 [file ijms-20-05162-s001.zip › Supplementary/Supplemental Figure 1.pdf]

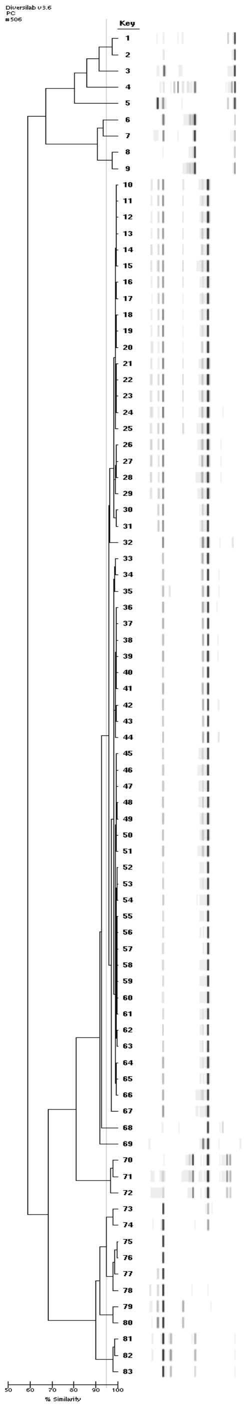

rep-PCR Typing	ST131	Phylo-genetic group	CH typing	Rx	CTX-M	LVFX susceptibility	QRDRs mutations[b] (number of mutations
A	non-ST131	D	88-145		CTX-M15	S	SDSE(0)
A	ST131	B2	40-30	non-Rx	CTX-M14	R	LNIV(4)
B	non-ST131	D	88-138		CTX-M55	S	SDSE
C	non-ST131	D	88-58		CTX-M2	R	LNIV(4)
D	non-ST131	D	4-27		CTX-M14	R	LNIE(3)
E	non-ST131	D	35-27		CTX-M14	R	LNIE(3)
F	non-ST131	D	26-5		CTX-M14	S	LDIE(2)
G	non-ST131	D	35-27		CTX-M14	S	LDSE(1)
G	non-ST131	B1	26-5		CTX-M14	R	LYIE(3)
H	ST131	B2	40-30	non-Rx	CTX-M27	R	LNIV(4)
H	ST131	B2	40-30	non-Rx	CTX-M14	R	LNIV(4)
H	ST131	B2	40-30	Rx	CTX-M15	R	LNIV(4)
H	ST131	B2	40-30	non-Rx	CTX-M14	R	LNIV(4)
H	ST131	B2	40-30	non-Rx	CTX-M14	R	LNIV(4)
H	ST131	B2	40-30	non-Rx	CTX-M14	R	LNIV(4)
H	ST131	B2	40-30	non-Rx	CTX-M27	R	LNIV(4)
H	ST131	B2	40-30	non-Rx	CTX-M27	R	LNIV(4)
H	ST131	B2	40-30	non-Rx	CTX-M14	R	LNIV(4)
H	ST131	B2	40-30	non-Rx	CTX-M14	R	LNIV(4)
H	ST131	B2	40-30	Rx	CTX-M15	R	LNIV(4)
H	ST131	B2	40-30	non-Rx	CTX-M27	R	LNIV(4)
H	ST131	B2	40-30	non-Rx	CTX-M14	R	LNIV(4)
H	ST131	B2	40-30	non-Rx	CTX-M14	R	LNIV(4)
H	ST131	B2	40-30	non-Rx	CTX-M27	R	LNIV(4)
H	ST131	B2	40-30	non-Rx	CTX-M15+M27	R	LNIV(4)
H	ST131	B2	40-30	non-Rx	CTX-M27	R	LNIV(4)
H	ST131	B2	40-30	Rx	CTX-M15	R	LNIV(4)
H	ST131	B2	40-30	non-Rx	CTX-M27	R	LNIV(4)
H	ST131	B2	40-30	Rx	CTX-M15	R	LNIV(4)
H	ST131	B2	40-41		CTX-M27	S	LDSE(1)
H	ST131	B2	40-41		CTX-M2G	S	LDSE(1)
H	ST131	B2	40-30	non-Rx	CTX-M27	R	LNIV(4)
H	ST131	B2	40-30	non-Rx	CTX-M27	R	LNIV(4)
H	ST131	B2	40-30	non-Rx	CTX-M14	R	LNIV(4)
H	ST131	B2	40-30	non-Rx	CTX-M15	R	LNIV(4)
H	ST131	B2	40-30	Rx	CTX-M15	R	LNIV(4)
H	ST131	B2	40-30	non-Rx	CTX-M14	R	LNIV(4)
H	ST131	B2	40-41		CTX-M2G	S	LDSE(1)
H	ST131	B2	40-30	Rx	CTX-M15	R	LNIV(4)
H	ST131	B2	40-30	non-Rx	CTX-M14	R	LNIV(4)
H	ST131	B2	40-30	non-Rx	CTX-M27	R	LNIV(4)
H	ST131	B2	40-30	Rx	CTX-M15	R	LNIV(4)
H	ST131	B2	40-30	non-Rx	CTX-M27	R	LNIV(4)
H	ST131	B2	40-41		CTX-M2G	S	LDSE(1)
H	ST131	B2	40-30	non-Rx	CTX-M14	R	LNIV(4)
H	non-ST131	B2	14-64		CTX-M27	R	LNIE(3)
H	ST131	B2	40-30	non-Rx	CTX-M14	R	LNIV(4)
H	ST131	B2	40-89		CTX-M14	S	LDSE(1)
H	ST131	B2	40-30	non-Rx	CTX-M14	R	LNIV(4)
H	ST131	B2	40-30	non-Rx	CTX-M14	R	LNIV(4)
H	ST131	B2	40-41		CTX-M55	S	LDSE(1)
H	ST131	B2	40-30	Rx	CTX-M15	R	LNIV(4)
H	ST131	B2	40-30	non-Rx	CTX-M14	R	LNIV(4)
H	ST131	B2	40-30	non-Rx	CTX-M14	R	LNIV(4)
H	ST131	B2	40-30	non-Rx	CTX-M14	R	LNIV(4)
H	ST131	B2	40-30	non-Rx	CTX-M27	R	LNIV(4)
H	ST131	B2	40-30	non-Rx	CTX-M14	R	LNIV(4)
H	ST131	B2	40-30	Rx	CTX-M15	R	LNIV(4)
H	ST131	B2	40-30	Rx	CTX-M15	R	LNIV(4)
H	ST131	B2	40-30	non-Rx	CTX-M14	R	LNIV(4)
H	ST131	B2	40-30	non-Rx	CTX-M14	R	LNIV(4)
H	ST131	B2	40-30	Rx	CTX-M15	R	LNIV(4)
H	ST131	B2	40-30	Rx	CTX-M15	R	LNIV(4)
H	ST131	B2	40-30	non-Rx	CTX-M14	R	LNIV(4)
H	ST131	B2	40-30	non-Rx	CTX-M14	R	LNIV(4)
H	ST131	B2	40-30	Rx	CTX-M15	R	LNIV(4)
H	ST131	B2	40-30	non-Rx	CTX-M14	R	LNIV(4)
I	non-ST131	B2	13-447		CTX-M27	S	LDSE(1)
J	non-ST131	B2	147-26		CTX-M2	S	SDSE(0)
K	non-ST131	D	100-96		CTX-M55	S	SDSE(0)
K	non-ST131	D	100-96		CTX-M55	S	SDSE(0)
K	non-ST131	D	100-96		CTX-M14	S	SDSE(0)
L	non-ST131	B2	14-64		CTX-M27	R	LNIE(3)
L	ST131	B2	40-30	Rx	CTX-M15	R	LNIV(4)
L	ST131	B2	40-30	non-Rx	CTX-M14	R	LNIV(4)
L	non-ST131	B2	14-30		CTX-M14	S	SDSE(0)
L	non-ST131	B2	14-64		CTX-M55	R	LNIE(3)
L	non-ST131	B2	14-64		CTX-M27	R	LNIE(3)
M	non-ST131	B2	14-64		CTX-M14	R	LNIE(3)
M	non-ST131	B2	14-64		CTX-M14	R	LNIE(3)
N	non-ST131	D	26-65		CTX-M3	R	LNIE(3)
N	non-ST131	D	26-65		CTX-M14	R	LNIE(3)
N	non-ST131	D	26-5		CTX-M14	S	LDSE(1)

Supplemental Figure 1. Dendrogram of repetitive-sequence-based PCR typing with 95% simirity level for 83 isolates of extended-spectrum β-lactamase (ESBL)-producing *Escherichia coli*. Sequence type (ST). LVFX Susceptibility was S (Susceptible) and R (Resistant). Substitution of QRDRs was expressed by amino acids in the order of 83 Ser (S) or Leu (L) and 87 Asp (D) or Asn (N) or Tyr (Y) in *gyrA*, and 80 Ser (S) or Ile (I) and 84 Glu (E) or Glu (E) in *parC* from the left (SDSE is wild-type).
